# miRNAs and Biomarkers in Testicular Germ Cell Tumors: An Update

**DOI:** 10.3390/ijms22031380

**Published:** 2021-01-30

**Authors:** Marco De Martino, Paolo Chieffi, Francesco Esposito

**Affiliations:** 1 Istituto di Endocrinologia ed Oncologia Sperimentale-CNR c/o Dipartimento di Medicina Molecolare e Biotecnologie Mediche, Università degli Studi di Napoli “Federico II”, Napoli 80131, Italy; marco.demartino2@unina.it (M.D.M.); francesco.esposito2@unina.it (F.E.); 2 Dipartimento di Medicina di Precisione, Università della Campania “L. Vanvitelli”, 80138 Napoli, Italy; 3 Dipartimento di Psicologia, Università della Campania “L. Vanvitelli”, 81100 Caserta, Italy

**Keywords:** testicular germ cell tumors, microRNA, biomarker

## Abstract

Testicular germ cell tumors (TGCTs) are the leading form of solid cancer and death affecting males between the ages of 20 and 40. Today, their surgical resection and chemotherapy are the treatments of first choice, even if sometimes this is not enough to save the lives of patients with TGCT. As seen for several tumors, the deregulation of microRNAs (miRNAs) is also a key feature in TGCTs. miRNAs are small molecules of RNA with biological activity that are released into biological fluids by testicular cancer cells. Their presence, therefore, can be detected and monitored by considering miRNAs as diagnostic and prognostic markers for TGCTs. The purpose of this review is to collect all the studies executed on miRNAs that have a potential role as biomarkers for testicular tumors.

## 1. Introduction

One of the most recurrent tumors in adolescent and young adults with the highest incidence between the ages of 20 and 40 years is testicular cancer [1,2]. Different testicular tumors require histological analyses for their therapies and prognosis. Testicular tumors are classified and divided into different subgroups by the International Agency for Research in Cancer of the World Health Organization (WHO). Other than sex cord-stromal tumors (Sertoli cell tumor, Leydig cell tumor), testicular germ cell tumors (TGCTs) occupy the main clinical role considering their frequent occurrence and the possibility of curing them, even when metastases are present, thanks to the introduction of cisplatin-based chemotherapy [3,4,5]. Among all genitourinary carcinomas [6], TGCTs have doubled their incidence because of unidentified environmental factors, undescended testes and positive family history [5]. In spite of the good results obtained with cisplatin treatment, numerous short- and long-term toxicity effects, such as thromboembolism, cardiovascular toxicity and neutropenic complications, are still reported [7,8,9,10]. Once patients tolerate risk-adapted chemotherapy, high cure rates can be achieved in aged patients with metastatic TGCT, as well as in younger patients [10,11]. Even though clinical examination is the diagnostic basis (scrotal ultrasound and high-resolution computed tomography), serum tumor markers maintain their clinical relevance. For instance, when there are suspected cases of testicular cancer, α-fetoprotein (AFP), the β-subunit of chorionic gonadotropin (β-hCG), as well as Lactate dehydrogenase (LDH), are analyzed [12]. In particular, β-hCG and AFP permit us to define TGCT diagnosis, histology, classification and prognosis. These markers are also used to monitor patients during active surveillance and for disease recurrence. Unfortunately, false positive or negative results can always arise [13,14]. Consequently, many studies have been conducted to find and utilize new tumor molecular markers with higher specificity and sensitivity. A new interesting class of tumor molecular markers is blood-circulating miRNAs. miRNAs are 19–22 nucleotides, in a single stranded shape, that thanks to a specific sequence act as post-transcriptional regulators modulating gene expression. miRNA genes are transcribed by RNA polymerase II, which generates a long primary transcript called pri-MiRNA [15]. Subsequently, the miRNA maturation process involves two main steps engaging RNase-III enzymes and double-stranded RNA-binding domain (dsRBD) proteins [15]. The nuclear RNase III-type enzyme Drosha is responsible for the production of a pre-miRNA, a hairpin precursor consisting of around 70 nt, through the maturation of the pri-miRNA. Then, the pre-miRNA is exported to the cytoplasm, where the RNase III protein Dicer processes it into an unstable miRNA duplex structure. Finally, the RNA-induced silencing complex (RISC) loads the less stable strand of the duplex into a nuclease complex that is able to regulate protein expression [15].

Indeed, the miRNA cluster 371–373, but also miRNAs such as miR-223-3, miR-449, miR-383, miR-514a-3p, miR-199a-3p and miR-214, have been proposed as new potential tumor markers [16]. So, the study and the clinical application of these new tumor markers is very interesting for many fields of research worldwide [17]. In this review, the clinical importance of previously offered tumor markers is debated and compared with new serological markers and detection methods, such as the quantification of miRNAs, so as to underline the new diagnostic options.

## 2. Novel TGCTs Biomarkers

### 2.1. HMGA Protein Family

The HMGA proteins, meaning proteins with high electrophoretic mobility, are made up of the proteins HMGA1a and HMGA1b, encoded via alternative splicing by the same gene *HMGA1*, and by the HMGA2 protein, encoded by the *HMGA2* gene [18,19]. Their main feature is that of possessing domains, called AT-hooks, that are able to bind DNA at the level of regions rich in adenine and thymine residues [19,20]. Although HMGA proteins participate in the transcriptional regulation of numerous genes, they are not transcriptional factors but chromatin architectural factors, with the ability to form protein complexes at the level of gene promoters and enhancers. The proteins HMGA1 and HMGA2 are strongly expressed during embryogenesis, while their expression becomes almost zero or completely absent in adult tissues. Nevertheless, their expression has been found to be upregulated in several human tumors, and it has also been attributed a causal role in neoplastic transformation [21,22]. Indeed, in vitro and in vivo experiments have clearly demonstrated that HMGA proteins are oncogenes. For example, both hmga1 and hmga2 are capable of transforming rat and murine fibroblasts, and *hmga1* or *hmga2* transgenic mice develop pituitary adenomas and NK-T lymphomas [19].

In the past, we have shown that HMGA1 is expressed in spermatogonia and primary spermatocytes (mitotic cells), whereas HMGA2 is expressed in secondary spermatocytes and spermatids (meiotic cells) [23]. In support of this, we have concluded that the spermatogenesis differentiation program in *hmga2* knock-out mice is strongly compromised. In addition, we have shown that in the event of a controversial TGCT diagnosis, HMGA proteins can be considered an advantageous diagnostic tool [24]. In fact, we have reported that HMGA proteins are expressed depending on the differentiation state of TGCTs: in pluripotential embryonal carcinoma cells, HMGA1 and HMGA2 have been reportedly overexpressed, in mature adult tissue of teratoma areas both proteins have not been detected, in seminomas just HMGA1 have been found to be overexpressed, and in yolk sac tumors just HMGA2 [24].

### 2.2. PATZ

PATZ1 is a transcriptional repressor that, thanks to its POZ domain, binds to the promoters of numerous genes, regulating their basal activity. In order to study its biological role, a knock-out mouse model for the PATZ1 gene was generated [25]. Interestingly, male patz1 −/− mice were infertile, highlighting a key role for this gene in spermatogenesis. Indeed, adult mice showed just a few apoptotic spermatocytes, other than a lack of spermatids and spermatozoa, leading to an alteration of the normal tubular structure. By northern blot, western blot and immunohistochemistry assays, PATZ was found expressed in Sertoli cells and in the spermatogonia [26]. Considering that *PATZ1* is a tumor suppressor gene, its role in TGCTs has been investigated. Indeed, *PATZ1* tumor suppressor activity has been found to be impaired in TGCT because of its delocalization into the cytoplasm of cancer cells. This delocalization is likely due to the downregulation of the estrogen receptor β, a PATZ1-interacting molecular partner. In fact, it has been shown that PATZ1 interacts with ERβ in normal germ cells, while the downregulation of ERβ associates with PATZ1 and HMGA1 cytoplasmic delocalization in seminomas [27,28].

These data suggest a crucial role for PATZ1 in normal male gametogenesis, whereas its overexpression and delocalization could be connected to the promotion of TGCTs.

### 2.3. G Protein-Coupled Oestrogen Receptor (GPR30)

Oestrogens are key hormones playing roles in testis physiology. Indeed, the estrogen receptors α (ERα) and estrogen receptors β (ERβ) are strongly expressed in specific testis cells. Whereas ERβ is almost ubiquitous, ERα is more specific, being expressed in Leydig cells and in the epithelium of efferent ductules [29,30]. Recent studies have underlined the role of other estrogen mediators, such as G protein-coupled estrogen receptor 30 (GPR30). Indeed, GPR30 has been widely accepted as a key regulator of testis pathophysiology [31,32]. Interestingly, its expression has been found in normal human spermatogonia and in spermatocytes, as well as in seminoma samples [32]. Moreover, a strong GPR30 overexpression was found in human carcinoma in situ (CIS) and in seminomas where the ERβ levels were downregulated, thus negatively correlating. Furthermore, GPR30 is able to activate the ERK1/2 signaling pathway by binding to 17β-estradiol in the seminoma-derived cell line (TCam-2) [33]. Thus, ongoing studies are trying to use GPR30 as a biomarker for human seminoma, as well as to design novel therapies to halt TGCT development by targeting this receptor [32,33].

### 2.4. Aurora B Kinase

Very often, human cancer is induced by the alteration of several cell cycle regulators [34]. In TGCTs, one of the best-studied cell cycle regulators is Aurora B Kinase. Indeed, Aurora B, which is the predominant kinase during G2-M transition, is able to phosphorylate serine 10 of histone H3, a histone modification deeply involved in chromosome condensation, alignment and segregation. Moreover, Aurora B is a key regulator of the spindle check point and cytokinesis [34]. As a molecular marker, this kinase has been used to discriminate the different TGCT histotypes: Aurora B expression has been found in intratubular germ cell neoplasia (IGCNU), seminoma and embryonal carcinoma, whereas it is absent in teratoma and yolk sac tumors (YSTs) [35,36,37]. Furthermore, Aurora B represents a valuable target for therapy. Indeed, its inhibition is able to drastically reduce cell proliferation in two testis cell lines (GC1 and TCam-2) [38]. Several compounds are actually in early clinical evaluation to test their ability to block tumor growth on a wide panel of human cancer types.

### 2.5. microRNAs in TGCT: From the Pathogenesis to a Novel Tumoral Biomarker Role

In the last few years, more and more research groups have focused their studies on a novel class of short non-coding RNAs, which are transcribed but not translated: miRNAs [39]. This class is constituted of small RNA molecules of about 19–22 nucleotides that are able to bind the 3′unuranslated regions (UTRs) of several transcripts, leading to the degradation of the targeted mRNAs, or to block the protein translation [17,40]. Thus, miRNAs became key molecules involved in both physiological and pathological processes. In particular, cancer research has been revolutionized by miRNAs discovery, since the deregulation of several miRNAs has been involved in the pathogenesis of many human cancer types [41,42,43]. Indeed, this class of short non-coding RNAs is necessary for a variety of cell mechanisms such as cell cycle regulation, migration/invasion, drug resistance and immune response [44,45,46]. Given their importance in cancer phenomena, alterations of the miRNA signature in cancer cells vs. normal cells have been used to better determine the diagnosis, the prognosis and the response to cancer treatment in several human cancers [47]. More recently, miRNAs have been found also in the extracellular *milieu* with signal transduction functions. Indeed, their structure is highly stable in the blood stream, and they can be used for cell–cell communication in an autocrine, paracrine or endocrine manner [48]. Moreover, they can be released following cell death [49]. Thus, their abundance in serum and the relative easiness of detection makes them suitable also as potential circulating tumor markers.

### 2.6. miR-302/367 Cluster

The cell cycle of embryonic (ESC) and pluripotent stem cells (PSC) is in part regulated by the miR-302 cluster. In fact, miR-302 induces the fast proliferation of these cells interacting with promoters and inhibitors of genes that regulate the cell cycle [50].

Moreover, the miR-302 cluster influences cell cycle progression by modifying histone methylations and the Akt/PKB pathway [51]. Akt oncogene expression has been found to be suppressed by miR-302 in teratomas. In this context, OCT4, a pluripotent transcription factor, is not still inhibited by Akt. On the contrary, Akt upregulation, as a result of miR-302 downregulation, causes low OCT4 expression. Indeed, OCT4 overexpression is necessary for teratoma formation and promotes PSCs pluripotent skills. It has been demonstrated that miR-302 accelerates the cell cycle switch from the G1 to the S phase, inhibiting cyclin-dependent kinase (CDK) 2 and 4 [52,53]. The blood of TGCT patients contains high levels of miR-302, whereas this is downregulated in liver, stomach and colon cancer [54,55,56,57]. Interestingly, the levels of miR-302a-3p, miR-302b-3p and miR-302c-3p diminished in TGCT cell lines after cisplatin treatment [52].

Sprouty RTK Signaling Antagonist 4 (SPRY4), which alters the PI3K/Akt signaling pathway, has been found overexpressed in TGCT. A possible role for miR-302 in the growth of TGCTs is proven by the fact that its inhibition results in a SPRY4 suppression that in turn decreases TGCT growth and invasion [57]. In conclusion, as demonstrated by Murray et al., miR-302 upregulation has been detected in all blood samples analyzed from TGCT patients [58].

Strictly associated with miR-302, there is miR-367. Whereas their sequences have some differences, they share several mRNAs as targets [51]. Similar to miR-302, miR-367 is able to regulate cancer-related pathways and cell cycles [59]. miR-367-3p serum levels were used to determine TGCT tumoral stage. Interestingly, miR-367-3p was strongly upregulated in TGCT patients compared to healthy ones. Moreover, its serum levels were decreased in Stage I and Stage II TGCTs compared with Stage III, where they strongly diminished after orchiectomy. However, miR-367-3p serum levels failed to detect teratoma and possible TGCT precursors, such as germ cell neoplasia in situ (GCNIS), or benign lesions [60,61]. Although some positive results were achieved by using miR-367-3p as a marker of chemotherapy-resistant germinal tumors [62], several other studies reported that miR-371-3p has a higher sensitivity as a marker of TGCT disease and/or relapse [63,64], thus further studies are needed.

### 2.7. miR-371-3 Cluster

The miR-371-3 locus represents the main miRNA cluster involved in TGCT tumorigenesis. It is settled on chromosome 19, and consists of a polycistronic miRNA transcript (pri-miR-371-3) that is then cleaved in four different molecules: miR-371, miR-372, miR-373 and miR-373* [16,65]. The upregulation of this cluster has been found in a number of human cancers, since it is involved in cell proliferation [66,67,68], drug resistance [69], migration and invasion [70,71]. In TGCTs, this cluster has been found to be mainly overexpressed. Interestingly, it has been reported that miR-372 and miR-373 are able to reduce the expression of the Large Tumor Suppressor Kinase 2 (LATS2), thus inhibiting p53 activity and triggering the accumulation of DNA mutations [72,73]. Moreover, Zhou et al. reported that the miR-371-3 cluster may activate the Wnt/β-catenin pathway and downregulate the Dickkopf-1 (DKK1) protein, thus sustaining cell proliferation and the invasion properties of cancer cells [74]. Although many papers have underlined the molecular mechanisms by which this cluster participates in tumor transformation, only miR-371a-3p has been extensively analyzed as a TGCT biomarker in follow-up, staging and diagnosis, and to estimate TGCT prognosis. Indeed, miR-371a-3p has been reported also as a powerful diagnostic tool able to discriminate various testicular histotypes. Briefly, the miR-371a-3p levels were assessed in the histological sections of different TGCT specimens by qRT-PCR. As expected, miR expression levels were found to be increased in TGCT samples compared to normal testis tissues, and TGCTs derived from GCNIS showed higher miR-371a-3p levels than non-GCNIS tumors. Importantly, seminomas patients displayed the highest miR-371a-3p expression, followed by embryonic carcinomas, teratomas and yolk sac tumors (Table 1) [75]. Furthermore, miR-371a-3p seems to be the first miRNA suitable for assessing the presence of metastatic disease and monitoring treatment success in cisplatin-treated TGCTs patients [16].

### 2.8. miR-517/519

Near the miR-371-373 cluster, there are three miRNAs, miR-517a-3p, miR-519a-3p, and miR-519c-3p, that belong to the same chromosome miRNA cluster (C19MC) [76]. In several tumors, it has been found that the upregulation of these three miRNAs is responsible for their enhanced migration, invasion, and poor overall survival [77,78]. Flor et al. studied the expressions of miR-517a-3p, miR-519a-3p, and miR-519c-3p in TGCTs. They found the same or a lower expression of these three miRNAs in Stage I seminoma and teratoma mixed tumors in comparison with normal testis. On the contrary, high miRNAs expression was detected in non-seminomatous and Stage III mixed tumors.

These data sustain the role of miR-517a-3p, miR-519a-3p and miR-519c-3p as a novel tumor marker for advanced-stage and non-seminomatous tumors. Interestingly, following tumor resection, their levels in the serum were reduced [79].

### 2.9. miR-223-3p

The scientific literature reports that miR-223-3p has been found to be deregulated in gastric and esophageal tumors, as well as in acute T-cell lymphoblastic leukemia [80,81,82]. In prostate cancer (PCa) tissue, miR-223-3p expression is recurrently reduced in comparison to the normal tissues, where miR-223-3p works as a tumor suppressor preventing cancer cell migration and invasion [83]. Moreover, miR-223-3p is upregulated in TGCTs with respect to normal testis. In fact, the Cancer Genome Atlas (TCGA) database and published data set reported a higher miR-223-3p expression in tumors than in normal testes. Additionally, a negative correlation was found between miR-223-3p and FBXW7 mRNA expression levels. In TGCT tumors, miR-223-3p upregulation provokes the inhibition of apoptosis mediated by the F-box/WD repeat-containing protein 7 (FBXW7), which belongs to a protein complex acting as a tumor suppressor that sustains the degradation of oncoproteins, including c-Myc, cyclin E, MCL-1, c-JUN, NFκB2 and Notch1 [84]. Additionally, the ectopic expression of the full-length coding sequence of FBXW7 rescued the cell growth and apoptosis mediated by miR-223-3p. As demonstrated in gastric cancer [85], miR-223-3p may influence chemotherapeutic agents’ sensitivity in TGCTs. Finally, more studies are needed to use miR-223-3p as a tumor marker.

### 2.10. miR-449

A main role in spermatogenesis has been found for miR-449. In fact, even though miR-34b/c and miR-449 are not necessary for male fertility, mutual ablation impedes normal spermatogenesis and provokes male infertility [86].

The transcription factor E2F is able to regulate miR-449a and miR-449b transcription [87]. Cell cycle and apoptosis are strictly modulated by E2F. miR-449a can counteract the progression of the cell cycle by reducing the expression of Cyclin-Dependent Kinase 6 (CDK6) [88]. miR-449a/b structurally calls to mind the miRNA 34 family, which is induced by p53. Consistently with a supposed tumor-suppressive role, miR-449a, along with miR-34a, inhibited proliferation and promoted apoptosis by at least partially p53-independent mechanisms. In testicular cancer, miR-449a expression was low or absent, whereas it is expressed in healthy testicular tissue. This is likely due to retinoblastoma mutations (pRB), which are not still able to bind to E2F. Therefore, a low amount of E2F brings about a downregulation of miR-449a that promotes cell cycle progression in tumor cells [89].

### 2.11. miR-383

miR-383 regulates the different biological activities of TGCTs, such as apoptosis, proliferation and cell cycle regulations [90,91]. It has been found to be overexpressed in embryonic carcinomas, while it is downregulated in infertile men. miR-383 binds to interferon regulatory factor-1 (IRF1), leading to its downregulation. Decreased IRF1 expression provokes the downregulation of its molecular partners involved in cell cycle regulation, such as cyclin D1, CDK2 and p21 [92]. As described for miR-449, miR-383 regulates the pRB protein by binding to IRF1 mRNA in embryonic carcinomas [90]. In embryonic carcinomas, the inhibition of H2AX, the DNA damage marker, is likely due to miR-383 overexpression. This phenomenon increases the tumor’s sensitivity to cisplatin and suggests for miR-383 the role of potential target in embryonic carcinomas [93].

### 2.12. Let-7a and miR-26a

Let-7a and miR-26a act as tumor suppressor miRNAs in different human cancer types [94,95,96]. Consistently, De Martino et al. showed that Let-7a and miR-26a were downregulated also in human seminoma. Moreover, they demonstrated that Let-7a and miR-26a were able to inhibit seminoma cell growth and mobility by inhibiting *HMGA1* levels [97].

Intriguingly, Let-7a and miR-26a’s inhibitory effects on seminoma cell growth could produce new understandings from therapeutic perspectives. In reality, new therapy approaches may restore the normal levels of Let-7a and miR-26a in seminomas by administering synthetic miRNAs [97].

## 3. Conclusions

**Figure 1:**
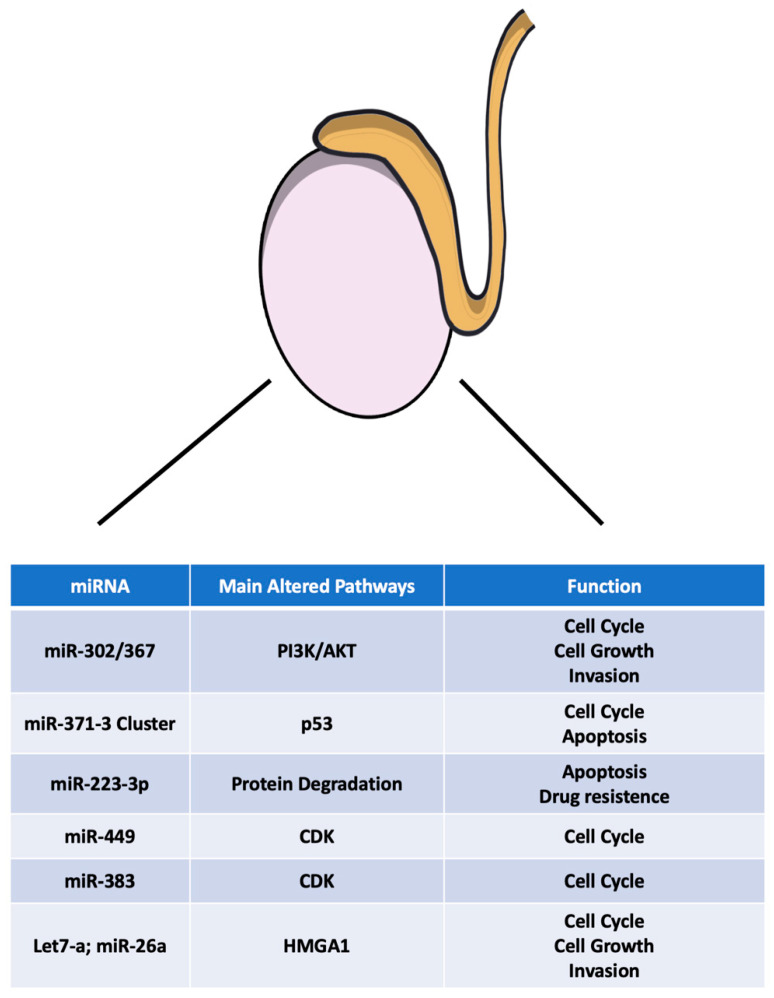
Main microRNAs deregulated in TGCTs. PI3K: Phosphoinositide 3-kinase; AKT: CDK: cyclin-dependent kinase.

This paper aimed to review the canonical and emerging tumoral markers for TGCT disease. Indeed, until now, several markers have been used in diagnosis and prognosis; however, novel molecules, such as miRNAs, may be of great potential as tumor markers or therapeutics, even though larger studies are needed (Figure 1). Indeed, the advantages of using miRNAs as tumoral markers are numerous. First of all, they are easy to obtain without severe damage for the patients. They can be simply collected from blood, urine and semen. Moreover, the current miRNAs detection methods, such as polymerase chain reaction (PCR), make circulating miRNAs more sensitive biomarkers in comparison with traditional biopsies and/or image examinations. Finally, the expression pattern of miRNAs gives the opportunity to assess the stage and progression of tumors in real time and in a dynamic manner, from tumorigenesis throughout the following progression. Therefore, miRNAs show a prospective capacity to serve as non-invasive cancer biomarkers for clinical application. They may be applied in many aspects, such as cancer screening, subtype classification and drug sensitivity prediction for treatment strategy selection.

However, numerous independent validation studies are still required to improve the use of miRNAs in clinical applications. A main contribution could come from the combination of miRNAs with other canonical biomarkers, which could also result in a comprehensive view of miRNAs, known and unknown, long non-coding RNAs (lncRNAs), circular-RNAs, and other ncRNAs.

## Figures and Tables

**Table 1:** miR-371a-3p levels as discriminative diagnostic tool.

	Seminoma	Embryonal Carcinoma	Teratoma	Yolk Sac Tumor	
				Pre-puberal	Post-puberal
miR-371a-3p	++++	+++	++	+	+
